# Identification of tapetum-specific genes by comparing global gene expression of four different male sterile lines in *Brassica oleracea*

**DOI:** 10.1007/s11103-015-0287-0

**Published:** 2015-02-25

**Authors:** Yuan Ma, Jungen Kang, Jian Wu, Yingguo Zhu, Xiaowu Wang

**Affiliations:** 1 Key Laboratory of the Ministry of Education for Plant Developmental Biology, College of Life Sciences, Wuhan University, Wuhan, 430072 China; 2 Institute of Vegetables and Flowers, Chinese Academy of Agricultural Sciences, Zhongguancun South Street 12, Beijing, 100087 China; 3 Vegetable Research Center, Beijing Academy of Agriculture and Forestry Sciences, Landianchang South Street 5, Beijing, 100081 China

**Keywords:** *Brassica oleracea*, Tapetum, Gene expression, Male sterility (MS), Microarray

## Abstract

The tapetum plays an important role in anther development by providing necessary enzymes and nutrients for pollen development. However, it is difficult to identify tapetum-specific genes on a large-scale because of the difficulty of separating tapetum cells from other anther tissues. Here, we reported the identification of tapetum-specific genes by comparing the gene expression patterns of four male sterile (MS) lines of *Brassica oleracea.* The abortive phenotypes of the four MS lines revealed different defects in tapetum and pollen development but normal anther wall development when observed by transmission electron microscopy. These tapetum displayed continuous defective characteristics throughout the anther developmental stages. The transcriptome from flower buds, covering all anther developmental stages, was analyzed and bioinformatics analyses exploring tapetum development-related genes were performed. We identified 1,005 genes differentially expressed in at least one of the MS lines and 104 were non-pollen expressed genes (NPGs). Most of the identified NPGs were tapetum-specific genes considering that anther walls were normally developed in all four MS lines. Among the 104 NPGs, 22 genes were previously reported as being involved in tapetum development. We further separated the expressed NPGs into different developmental stages based on the MS defects. The data obtained in this study are not only informative for research on tapetum development in *B. oleracea*, but are also useful for genetic pathway research in other related species.

**Electronic supplementary material:**

The online version of this article (doi:10.1007/s11103-015-0287-0) contains supplementary material, which is available to authorized users.

## Introduction

Anther development comprises both gametophyte and anther wall development (Engelke et al. 2002; Ge et al. 2010; Ma and Sundaresan 2010). The connection between these two relatively independent processes occurs in the tapetum, a layer of the anther wall. As the innermost of four somatic layers, the tapetum surrounds developing reproductive cells and plays an important role in the male fertility of pollen grains. Its secretory tissue provides proteins, lipids, and other nutrients for pollen development and exine formation (Mariani et al. 1990; Piffanelli et al. 1998).

Anther development is divided into two main phases: microsporogenesis and microgametogenesis, and these are further subdivided into 14 anther stages (Chang et al. 2011; Ma 2005; Sanders et al. 1999; Smyth et al. 1990). In this research, to simplify these complex developmental processes, we divided these anther stages it into four major stages according to anther developmental events: the sporogenesis cell stage, pollen mother cell stage, pre-tetrad stage, and post-tetrad stage. In the sporogenesis cell stage, the sporogenous cells, which give rise to pollen, are visible within locules of sectioned anthers (Goldberg et al. 1993; Scott et al. 1991, 2004), In the pollen mother cell stage, sporogenous cells develop into pollen mother cells (Owen and Makaroff 1995; Stevens and Murray 1981), In the pre-tetrad stage, microspore mother cells enter meiosis while the middle layer is crushed and degenerates. Tapetum becomes vacuolated and the anther undergoes a general increase in size. Tetrads of microspores are free within each locule when meiosis is completed in the tetrad stage. In the post-tetrad stage, the callose wall surrounding tetrads degenerates and individual microspores are released. Microspores generate an exine wall and become vacuolated (Sanders et al. 1999). After these four stages, tapetum degeneration is initiated. The tapetum undergoes generation, development, and apoptosis, providing enzymes for the release of microspores (Varnier et al. 2005). Eventually, all cell remnants are released into the locules, due to tapetal degeneration, and are integrated into the pollen wall as pollen coat material (Papini et al. 1999). This series of events is completed in a relatively short time, and the progression of stages involves changes in the expression levels of many genes. The identification of genes associated with tapetum-related processes helps build a solid foundation for studying the underlying molecular mechanisms of anther development.

Because of difficulties in separating the tapetum cell layer from other anther wall cell layers, the large-scale identification of tapetum cell-specific genes has not previously been achieved. Tapetum-specific genes have been identified by looking for male sterility (MS) mutants in *Arabidopsis thaliana*. Only a limited number of tapetum-specific genes were reported, these included *ACOS5*, *A6*, *LTP12*, *LAP5*, *TSM1*, *TAP35*, *TAP44*, and *A9* (Ariizumi et al. 2002; de Azevedo et al. 2009; Fellenberg et al. 2008; Hird et al. 1993; Kim et al. 2010; Ma et al. 2012; Paul et al. 1992). Unfortunately, it is not possible to reveal the whole picture of tapetum gene expression by identifying specific tapetum gene mutants one by one. A large number of MS mutants have been identified from natural and artificial mutations in *Brassica*. Hybridization of *A. thaliana* cDNA arrays against close relatives that have bigger anthers, such as *Brassica* species, is a good approach for studying the genome-wide expression of anther-specific genes in *Arabidopsis* (Amagai et al. 2003).

Pollen grains can be easily isolated, which allows genes expressed in pollen grains to be easily profiled. A number of *Arabidopsis* pollen grain transcriptomes have been reported by Becker et al. (2003), Pina et al. (2005). Furthermore, a previous pollen transcriptome study by (Honys and Twell 2003) identified 992 pollen-expressed mRNAs, nearly 40 % of which were detected specifically in pollen. They also (Honys and Twell 2004) developed specific spore isolation procedures for *Arabidopsis* at the pollen developmental stage, and used Affymetrix ATH1 genomic arrays to identify 13,977 male gametophyte-expressed mRNAs in all stages of microsporogenesis, 9.7 % (1,355) of which were male gametophyte specific. However, comparative studies using the tapetum to identify anther wall-specific genes have not been reported in multiple MS lines in which MS mutants occur at different stages of tapetum development. Non-pollen expressed genes (NPGs), are the genes remaining after the exclusion of pollen-specific expressed genes from the genes expressed specifically in the anther. This provides a narrow range for the identification of potential tapetum-specific expressed genes.

This study employs four types of *B. oleracea* MS lines: *Nigra* cytoplasm male sterility (NiCMS), Ogura cytoplasm male sterility (OguCMS), recessive male sterility (RGMS) and dominant male sterility (DGMS) (Kang et al. 2008; Fang et al. 2001). Each MS line has a distinct tapetum abortion phenotype and their abnormal characteristics appear successively during anther development. For the large-scale identification of tapetum-specific genes and to gain further insight into downstream cellular reactions of tapetum development, we compared the anther transcriptomes of the four types of *B. oleracea* MS lines through the heterologous hybridization of *B. oleracea* mRNA onto an *Arabidopsis* whole genome oligonucleotide microarray.

## Materials and methods

### Plant materials

Four *B. oleracea* MS lines which are different from types and origins were used in this study (Table 1) (Kao et al. 1992; Pearson 1972; Fang et al. 1984, 1995): Nigra cytoplasmic MS line NiCMS-803B, recessive MS line RGMS-802B, Ogura cytoplasmic MS line OguCMS-MsC-881, and dominant MS line DGMS-MsC-881, which were supplied by the Institute of Vegetables and Flowers, Chinese Academy of Agriculture Sciences. All four MS lines had been backcrossed to fertile parents for nine generations. All flower buds above the last opened flower of three flowering branches were collected from six MS plants and six corresponding control lines (MF; 803, 802, 881, and 881 K) during the full flowering stage for cytological observation and microarray experiments. All plants after vernalization were grown in a climate controlled greenhouse set at 70 % relative humidity with a 20/15 °C (12 h/12 h) day/night temperature regime for 35–40 days.

**Table 1:** The description of four male sterile lines in this study

MS materials	Types	Origins	Transfer methods	References
NiCMS	Cytoplasmic male sterile	*Brassica nigra*	Protoplast fusion	Pearson 1972
RGMS	Recessive male sterile	*Brassica oleracea*	Natural mutant	Fang et al. 1984
OguCMS	Cytoplasmic male sterile	*Raphanus sativus*	Protoplast fusion	Kao et al. 1992
DGMS	Dominant male sterile	*Brassica oleracea*	Natural mutant	Fang et al. 1995

### Cytological analysis using microscopes

Light microscopy and transmission electron microscopy (TEM) were used to investigate the cytological differences between the mutants and wild-type. Different sized buds were fixed overnight in 4 % glutaraldehyde with 200 mM phosphate buffer (pH 7.0) and then rinsed overnight in 200 mM phosphate buffer (pH 7.0). Next, buds were post-fixed in a solution containing 1 % osmium tetroxide for 2 h and washed in a dehydrated ethanol series for 30 min at each step (2 × 50, 60, 70, 80, 90, and 2 × 100 %). Dehydrated tissues were embedded in Spurr’s epoxy resin (Spurr 1969; Ted Pella, Redding, CA) for 3 days at 60 °C, and sectioned at 60–90 nm using a microtome (Leica Ultratome V, LKB, Bromma, Sweden). Anther transverse sections were stained in 4 % uranyl acetate for 20 min and in lead citrate for 3 min. All specimens were analyzed using TEM (H-8100, Hitachi, Tokyo, Japan). For the light microscopy analysis, buds were fixed overnight in FAA (50 % ethanol, 5.0 % glacial acetic acid, 3.7 % formaldehyde), dehydrated in a graded ethanol series (2 × 50, 60, 70, 85, 95, and 3 × 100 %), embedded in Spurr’s epoxy resin, and sectioned in 1 µm thick slices using a microtome. Anther transverse sections were stained in 1 % toluidine blue at 42 °C for 1–2 h and observed under a compound microscope (Olympus Model BH2, Tokyo, Japan).

### RNA preparation, probe labeling, and microarray hybridization

For total RNA isolation, all flower buds above the last opened flowers of three flowering branches from three MS and three MF plants were collected in duplicate and combined to reduce plant-to-plant variation. Total RNA was isolated using TRIzol following the manufacturer’s instructions (Invitrogen, Beijing, China). RNA was further purified using an RNeasy Mini kit (Qiagen China Co., Ltd., Shanghai, China) and the quality was checked using an Agilent 2100 bioanalyzer (Agilent Technologies China Co., Ltd., Shanghai, China) and RNA 6000 Nano kit (Agilent Technologies China Co., Ltd., Shanghai, China) before labeled cRNA was synthesized. Cy3- and Cy5-labeled cRNA was synthesized from 400 ng total RNA using a Low RNA Input Linear Amplification and Labeling kit Plus (Agilent Technologies China Co., Ltd., Shanghai, China) following manufacturer’s protocol. Swap labeling of the other MS and MF (three plant pools) RNAs was conducted for the replications. Labeled cRNA was hybridized onto a 22 K *Arabidopsis* oligo microarray (Agilent Technologies China Co., Ltd., Shanghai, China) using the In situ Hybridization kit Plus (Agilent Technologies China Co., Ltd., Shanghai, China).

### Data acquisition, normalization, and gene annotation analysis

Hybridized microarrays were scanned sequentially for Cy3- and Cy5-labeled probes with a laser scanner (G2655AA, Agilent Technologies China Co., Ltd., Shanghai, China) at a resolution of 10 µm and a PMT of 100. The intensities were normalized by linear LOWESS (Yang et al. 2002). The signal was considered positive when the signal/noise value was >5. To determine pollen stage specificity and co-expression information, our data were compared with array data from two other studies. The pollen transcriptome refers to the dataset from (Honys and Twell 2004), and the stamen transcriptome refers to the dataset from (Wellmer et al. 2004). Microsoft Office Excel (Excel 2010, Microsoft China Co., Ltd., Beijing, China) was used to manage and filter the microarray data. Differently expressed genes (DEGs) were functionally categorized based on the ontology annotation of the *Arabidopsis* genome from the Arabidopsis Information Resource (http://www.arabidopsis.org). Pollen expression type was determined based on the maximum expression value (MaxP) from the four pollen stages (uninucleate microspore, bicellular pollen, tricellular pollen, and mature pollen), the maximum expression value (MaxS) from seven sporophytic tissues (cotyledon, leaves, petiole, stems, roots, root hair zone, and suspension cell cultures), or the value of MaxP/MaxS according to the pollen transcriptome dataset (Honys and Twell 2004). Late pollen genes were defined as those expressed after microspore mitosis during male gametophyte development, which had continued transcript accumulation during pollen maturation (McCormick 1993).

### Reverse transcription-polymerase chain reaction (RT-PCR)

Total RNA was treated with RNase-free DNase (Promega China Co., Ltd., Beijing, China) to remove genomic DNA. RT-PCR reactions were conducted using first-strand cDNA synthesized from 2 μg total RNA with Superscript II Transcriptase (Invitrogen, Beijing, China) and a poly-dT_18_ primer (Takara, Tokyo, Japan). The cDNAs were then used as templates for RT-PCR with gene-specific primers designed based on reference sequences of *B. oleracea ssp. capitata* line 02–12 retrieved from BRAD (http://brassicadb.org/brad; Table 2). The *Translation Elongation Factor* gene *EF*-*1α* was used as a positive control togage the quantity of input cDNA among the different samples. The primers used are listed in Supplementary Table S5.

**Table 2:** The 104 non-pollen expressed genes (NPGs) from *Brassica oleracea* detected in this study

Gene ID	Group name	Gene description	Down-regulated ratio of fertility to sterility F/S				Expression pattern groups
			NiCMS	RGMS	OguCMS	DGMS	
AT1G01280	*CYP703A2*	Cytochrome P450, family 703, subfamily A, polypeptide 2	59.137	0.499	0.687	1.923	A
AT1G03390		HXXXD-type acyl-transferase family protein	3.376	0.819	0.309	0.931	A
AT1G27040		Major facilitator superfamily protein	3.664	2.117	2.985	1.008	A
AT1G36340	*UBC31*	Ubiquitin-conjugating enzyme 31	3.503	1.161	1.566	0.906	A
AT1G52560		HSP20-like chaperones superfamily protein	49.751	1.362	1.769	0.772	A
AT1G62940	*ACOS5*	Acyl-CoA synthetase 5	21.156	0.364	0.403	1.246	A
AT1G74310	*HSP101*	Heat shock protein 101	9.024	0.971	0.957	2.327	A
AT2G14540	*SRP2*	Serpin 2	8.913	0.378	0.857	2.331	A
AT2G26150	*HSFA2*	Heat shock transcription factor A2	4.045	0.955	1.087	2.105	A
AT2G31210		Basic helix-loop-helix (bHLH) DNA-binding superfamily protein	3.998	2.032	0.512	0.969	A
AT2G38240		2-oxoglutarate (2OG) and Fe(II)-dependent oxygenase superfamily protein	3.035	1.621	1.630	0.377	A
AT2G42940		Predicted AT-hook DNA-binding family protein	36.341	0.336	0.086	2.476	A
AT2G45630		d-isomer specific 2-hydroxyacid dehydrogenase family protein	3.502	1.118	1.133	1.450	A
AT3G05780	*LON3*	lon protease 3	3.075	0.311	0.330	0.898	A
AT3G09640	*APX2*	Ascorbate peroxidase 2	7.165	1.048	2.410	0.905	A
AT3G13220	*WBC27*	ABC-2 type transporter family protein	15.681	0.560	0.704	1.188	A
AT3G48540		Cytidine/deoxycytidylate deaminase family protein	3.199	1.013	0.845	0.817	A
AT3G52130		Bifunctional inhibitor/lipid-transfer protein/seed storage 2S albumin superfamily protein	9.011	0.495	0.245	0.654	A
AT4G14080	*MEE48*	O-Glycosyl hydrolases family 17 protein	85.174	0.322	0.406	1.871	A
AT4G20800		FAD-binding Berberine family protein	3.556	0.216	0.478	0.496	A
AT4G34850	*LAP5*	Chalcone and stilbene synthase family protein	33.679	0.347	0.524	2.273	A
AT5G02490	*Hsp70*-*2*	Heat shock protein 70 (Hsp 70) family protein	3.638	0.991	0.994	1.583	A
AT5G03800	*EMB1899*	Pentatricopeptide repeat (PPR) superfamily protein	3.957	0.603	0.398	1.511	A
AT5G07230		Bifunctional inhibitor/lipid-transfer protein/seed storage 2S albumin superfamily protein	38.418	1.387	1.473	1.206	A
AT5G15250	*FTSH6*	FTSH protease 6	6.159	0.940	1.604	0.937	A
AT5G56110	*MYB80*	myb domain protein 103	11.942	0.297	0.310	1.641	A
AT5G59330		Bifunctional inhibitor/lipid-transfer protein/seed storage 2S albumin superfamily protein	3.228	0.863	1.869	0.416	A
AT5G66110	*HIPP27*	Heavy metal transport/detoxification superfamily protein	4.063	0.950	1.877	1.133	A
AT1G06170		Basic helix-loop-helix (bHLH) DNA-binding superfamily protein	21.653	3.183	0.946	1.042	B
AT1G26780	*MYB117*	myb domain protein 117	5.846	3.004	2.601	1.306	B
AT1G61070	*PDF2.4*	low-molecular-weight cysteine-rich 66	19.633	6.853	1.030	0.804	B
AT2G16910	*AMS*	Basic helix-loop-helix (bHLH) DNA-binding superfamily protein	66.945	4.721	0.651	1.476	B
AT3G13890	*MYB26*	myb domain protein 26	4.193	5.225	2.197	0.996	B
AT5G09970	*CYP78A7*	Cytochrome P450, family 78, subfamily A, polypeptide 7	27.337	10.050	0.731	0.837	B
AT5G59720	*HSP18.2*	Heat shock protein 18	61.107	3.567	2.700	1.710	B
AT1G06260		Cysteine proteinases superfamily protein	73.654	73.422	81.181	1.410	C
AT1G06990		GDSL-like Lipase/Acylhydrolase superfamily protein	35.896	27.959	28.634	1.008	C
AT1G09550		Pectinacetylesterase family protein	10.316	7.484	7.553	2.254	C
AT1G61110	*NAC025*	NAC domain containing protein 25	100.000	42.366	100.000	1.177	C
AT1G66570	*SUC7*	Sucrose-proton symporter 7	12.623	5.220	8.168	2.813	C
AT1G68190		B-box zinc finger family protein	9.385	3.859	4.895	1.220	C
AT1G71160	*KCS7*	3-ketoacyl-CoA synthase 7	40.698	7.545	4.751	1.043	C
AT1G74540	*CYP98A8*	Cytochrome P450, family 98, subfamily A, polypeptide 8	66.968	10.829	45.078	1.006	C
AT1G75930	*EXL6*	Extracellular lipase 6	41.703	29.272	31.621	1.245	C
AT2G03850		Late embryogenesis abundant protein (LEA) family protein	95.261	66.233	85.170	1.376	C
AT2G23800	*GGPS5*	Geranylgeranyl pyrophosphate synthase 2	6.929	5.344	4.895	1.002	C
AT3G51590	*LTP12*	Lipid transfer protein 12	73.651	29.780	100.000	1.015	C
AT3G56380	*RR17*	Response regulator 17	3.141	3.041	3.287	0.988	C
AT5G14980		Alpha/beta-Hydrolases superfamily protein	11.521	5.466	5.219	1.233	C
AT5G38160		Bifunctional inhibitor/lipid-transfer protein/seed storage 2S albumin superfamily protein	85.213	3.937	4.407	0.819	C
AT5G57670		Protein kinase superfamily protein	5.306	7.763	6.793	2.440	C
AT1G30860		RING/U-box superfamily protein	11.670	7.714	15.767	12.143	D
AT1G60210		Unknown	10.368	4.913	10.225	5.278	D
AT2G47040	*VGD1*	Plant invertase/pectin methylesterase inhibitor superfamily	85.995	94.127	100.000	13.393	D
AT4G37960		Unknown	91.658	100.000	100.000	8.117	D
AT1G27720	*TAF4B*	TBP-associated factor 4B	0.600	1.203	0.317	1.100	OguCMS_UP
AT1G60500	*DRP4C*	Dynamin related protein 4C	0.460	0.735	0.236	0.942	OguCMS_UP
AT1G68640	*PAN*	bZIP transcription factor family protein	0.961	0.742	0.258	0.738	OguCMS_UP
AT2G23050	*NPY4*	Phototropic-responsive NPH3 family protein	0.760	0.777	0.216	0.888	OguCMS_UP
AT3G13960	*GRF5*	Growth-regulating factor 5	0.714	1.087	0.327	0.957	OguCMS_UP
AT3G19300		Protein kinase superfamily protein	0.692	0.820	0.252	0.949	OguCMS_UP
AT4G10640	*IQD16*	IQ-domain 16	0.411	0.736	0.280	0.835	OguCMS_UP
AT4G29980			2.059	0.561	0.221	0.826	OguCMS_UP
AT5G13170	*SWEET15*	Senescence-associated gene 29	1.399	0.943	0.297	0.678	OguCMS_UP
AT5G26140	*LOG9*	Putative lysine decarboxylase family protein	1.131	1.406	0.333	0.743	OguCMS_UP
AT5G41890		GDSL-like Lipase/Acylhydrolase superfamily protein	1.215	1.296	0.303	0.813	OguCMS_UP
AT5G42120		Concanavalin A-like lectin protein kinase family protein	0.763	0.724	0.168	0.837	OguCMS_UP
AT5G63390		O-fucosyltransferase family protein	0.548	0.840	0.334	1.347	OguCMS_UP
AT1G26400		FAD-binding Berberine family protein	0.977	0.093	0.206	0.381	RGMS & OguCMS_UP
AT1G53990	*GLIP3*	GDSL-motif lipase 3	0.849	0.166	0.217	0.612	RGMS & OguCMS_UP
AT1G73050		Glucose-methanol-choline (GMC) oxidoreductase family protein	0.807	0.264	0.075	0.482	RGMS & OguCMS_UP
AT3G21660		UBX domain-containing protein	1.380	0.249	0.100	0.865	RGMS & OguCMS_UP
AT5G54060	*UF3GT*	UDP-glucose:flavonoid 3-o-glucosyltransferase	1.248	0.331	0.324	1.486	RGMS & OguCMS_UP
AT5G10880		tRNA synthetase-related/tRNA ligase-related	1.794	0.517	0.190	0.287	OguCMS & DGMS_UP
AT1G69500	*CYP704B1*	Cytochrome P450, family 704, subfamily B, polypeptide 1	80.639	5.991	0.789	1.331	
AT1G75890		GDSL-like Lipase/Acylhydrolase superfamily protein	11.565	3.048	8.453	1.224	
AT3G10600	*CAT7*	Cationic amino acid transporter 7	2.700	4.204	1.349	1.514	
AT3G15870		Fatty acid desaturase family protein	0.347	3.419	0.741	1.786	
AT1G64010		Serine protease inhibitor (SERPIN) family protein	20.572	0.279	1.114	3.002	
AT3G11980	*MS2*	Jojoba acyl CoA reductase-related male sterility protein	88.115	0.664	0.728	5.882	
AT2G13900		Cysteine/Histidine-rich C1 domain family protein	10.295	14.914	16.371	6.944	
AT1G28430	*CYP705A24*	Cytochrome P450, family 705, subfamily A, polypeptide 24	5.695	2.973	5.814	1.383	
AT2G14960	*GH3.1*	Auxin-responsive GH3 family protein	3.708	0.732	3.282	1.158	
AT3G27812		Unknown	18.284	0.542	43.198	1.023	
AT3G53290	*CYP71B30P*	Cytochrome P450, family 71, subfamily B, polypeptide 30 pseudogene	9.250	0.375	16.667	1.371	
AT3G55970	*JRG21*	Jasmonate-regulated gene 21	5.141	0.497	4.167	0.781	
AT3G56700	*FAR6*	Fatty acid reductase 6	16.177	0.353	100.000	1.551	
AT1G03170	*FAF2*	Protein of unknown function (DUF3049)	0.681	0.673	3.176	1.515	
AT1G15360	*WIN1*	Integrase-type DNA-binding superfamily protein	1.929	1.215	4.281	1.031	
AT1G19640	*JMT*	Jasmonic acid carboxyl methyltransferase	1.141	0.772	5.879	1.379	
AT1G30740		FAD-binding Berberine family protein	2.387	2.498	4.238	2.632	
AT2G19990	*PR*-*1*-*LIKE*	Pathogenesis-related protein-1-like	1.504	2.561	6.212	5.556	
AT2G21220		SAUR-like auxin-responsive protein family	1.133	1.327	4.282	1.002	
AT2G23570	*MES19*	Methyl esterase 19	1.984	0.226	4.238	0.509	
AT2G30310		GDSL-like Lipase/Acylhydrolase family protein	2.397	1.417	3.256	1.043	
AT3G10570	*CYP77A6*	Cytochrome P450, family 77, subfamily A, polypeptide 6	1.852	1.168	3.098	0.842	
AT3G57510	*ADPG1*	Pectin lyase-like superfamily protein	1.774	1.043	5.391	1.049	
AT4G16000			0.601	0.850	4.465	0.886	
AT4G37950		Rhamnogalacturonate lyase family protein	2.729	1.904	3.469	2.782	
AT5G62320	*MYB99*	myb domain protein 99	30.564	0.958	4.055	1.460	
AT1G13150	*CYP86C4*	Cytochrome P450, family 86, subfamily C, polypeptide 4	77.360	21.613	43.020	1.065	
AT3G59440		Calcium-binding EF-hand family protein	1.879	4.605	3.068	1.951	
AT4G12410		SAUR-like auxin-responsive protein family	1.413	3.016	8.257	0.898	
AT4G23230	*CRK15*	Cysteine-rich RLK (RECEPTOR-like protein kinase) 15	13.486	7.928	8.071	1.337	

OguCMS_UP represents NPGs only up-regulated in the OguCMS line; RGMS & OguCMS_UP represents NPGs up-regulated in both the RGMS and OguCMS lines; OguCMS & DGMS_UP represents NPGs up-regulated in both the OguCMS and DGMS lines

A, B, C, and D present the expression order of down-regulated NPGs following the sequence of the abortive phenotypes appearance in the four male sterile (MS) lines of *Brassica oleracea* observed by light microscopy

### In situ hybridization

*Arabidopsis* Col-0 inflorescences were embedded in Paraplast (Sigma-Aldrich, Shanghai, China), sectioned at 8-μm thickness and mounted onto precharged slides. For sense and antisense probe synthesis, five coding regions of the NPGs, *MEE48*, *A9*, *CYP98A8*, *EXL6*, and *GGPS5*, resulting in 990-, 895-, 749-, 552-, and 656-bp DNA templates, were PCR amplified from flower cDNA using gene-specific forward and reverse primers. A T7 polymerase binding site was incorporated into the forward primer for sense probe amplification and in the reverse primer for antisense probe amplification. Digoxigenin-labeled probes were transcribed off the template using T7 polymerase (Roche, Shanghai, China). Probes were shortened to 200-bp fragments by limited carbonate hydrolysis, and then quantified and hybridized to slides. Tissue fixation, embedding, hybridization, and signal detection were performed as described by (Hooker et al. 2002).

## Results and discussion

### Cytological defects in the four *B. oleracea* MS lines

**Fig. 1:**
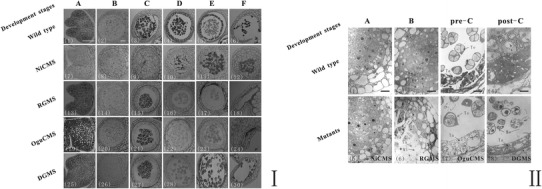
Light and transmission electron microscopy (TEM) micrographs of tapetums in wild-type and the four male sterile (MS) lines of *Brassica oleracea* at different developmental stages. **I** Comparisons of anther development between the wild-type (1–6) and four MS lines, NiCMS (7–12), RGMS (13–18), OguCMS (19–24), and DGMS (25–30), at different developmental stages, revealing that the principal cause of abnormal anther development in the four MS lines are defects in tapetum development, followed by abnormalities in microspore development. *Bar* = 20 μm. **II** Comparisons of tapetum development between the wild-type (1–4) and the four MS lines at different developmental stages (5–8), revealing that the sequence appearance and characteristics of tapetum cytological defects at the sporogenesis cell stage, microspore mother cell stage, pre-tetrad stage and post-tetrad stage. *Bar* = 2 μm. *A* the sporogenesis cell stage; *B* the microspore mother cell stage; *C* the early tetrad stage; *D* the uninucleate microspore stage; *E* the mature pollen stage; and *F* the dehiscence stage. *Sc* sporogenesis cell, *Ta* tapetum, *Ml* middle layer, *Mmc* microspore mother cell, *En* endothecium cell, *Ep* epidermis cell, *Te* tetrad, *Ms* microspore

**Fig. 2:**
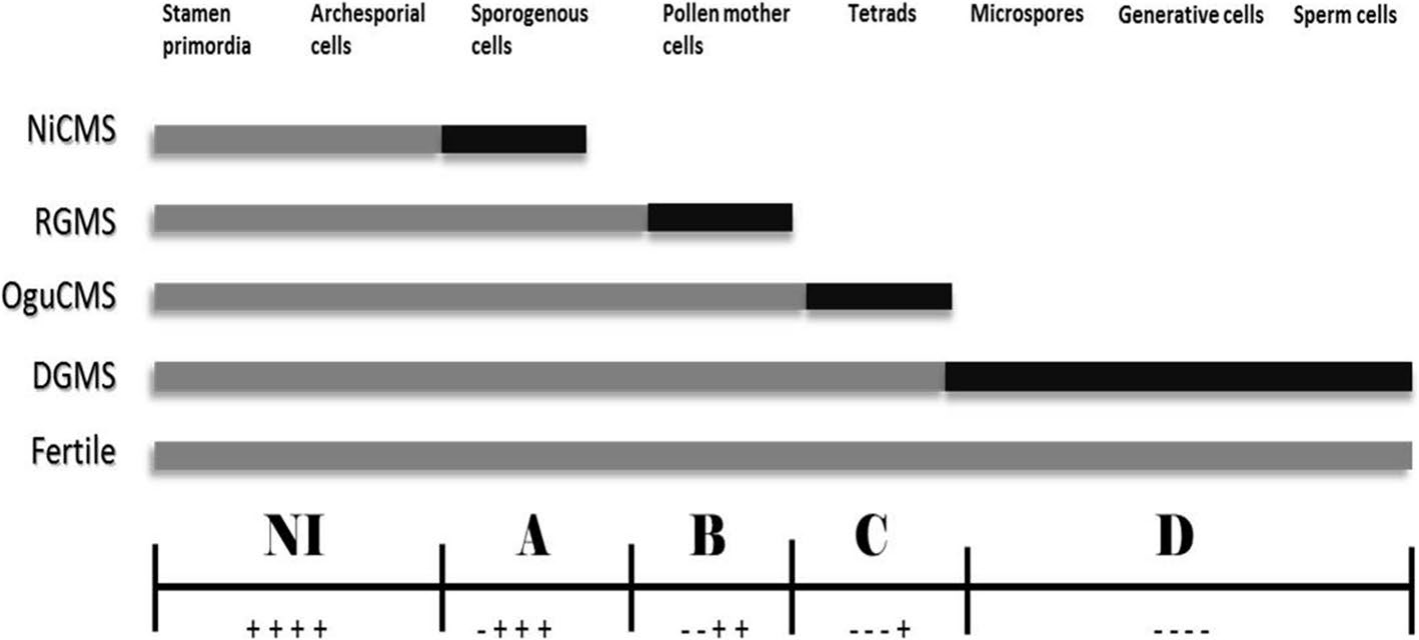
Sequential developmental interruption model for the four types of male sterile (MS) lines of *B. oleracea*. *Gray bar* represents the normal developmental stage. *Black bar* represents the male sterility stage. *NI* represents the stage in which the genes were not interrupted in all four MS lines. *A*, *B*, *C*, and *D* represent the expressed genes in different anther developmental stages, which were classified by the developmental interruptions of NiCMS, RGMS, OguCMS and DGMS, respectively. *Plus* represent up-regulated and *minus* represent down-regulated

**Fig. 3:**
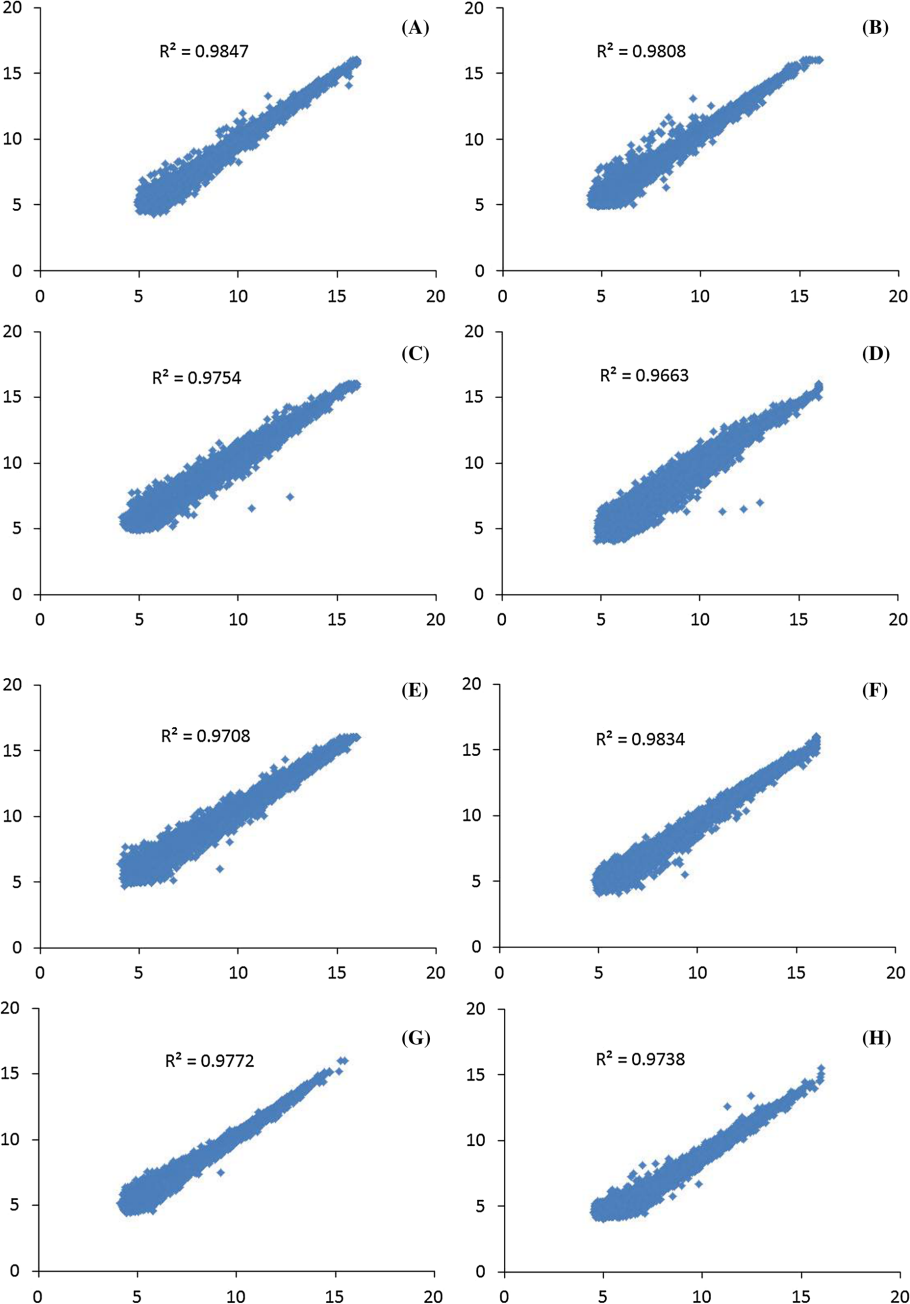
The correlation analysis between two swap replicates of the four male sterile (MS) lines of *Brassica oleracea* and corresponding control line (MF) buds. **a** NiCMS control; **b** NiCMS; **c** RGMS control; **d** RGMS; **e** OguCMS control; **f** OguCMS; **g** DGMS control; and **h** DGMS

**Fig. 4:**
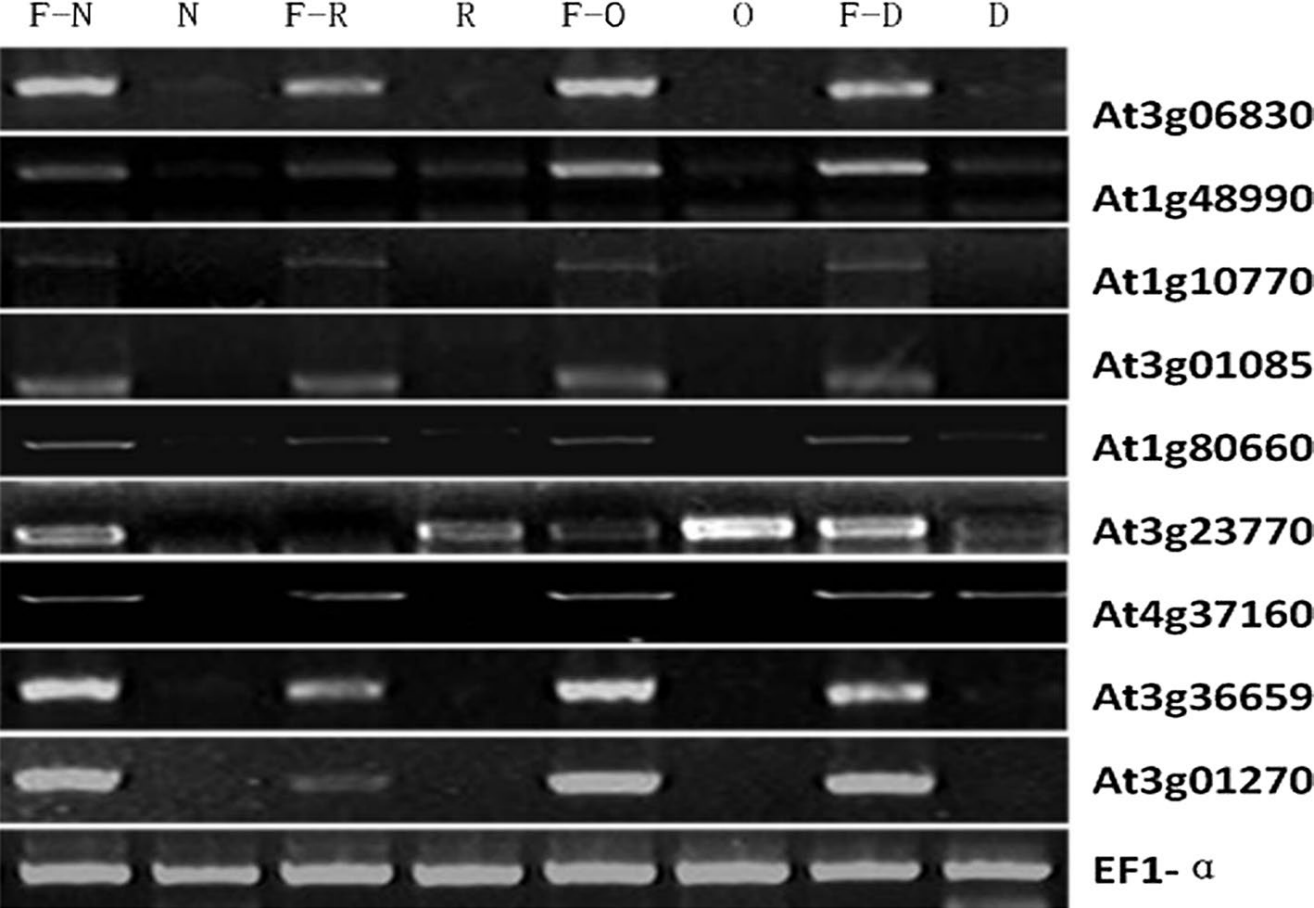
RT-PCR expression patterns of nine randomly selected genes. *N*, *R*, *O*, *D* represents the four male sterile (MS) lines of *Brassica oleracea*, NiCMS, RGMS, OguCMS, and DGMS. *F*–*N, F*–*R, F*–*O, F*–*D* represents the four MS lines’ corresponding control lines (MFs)

**Fig. 5:**
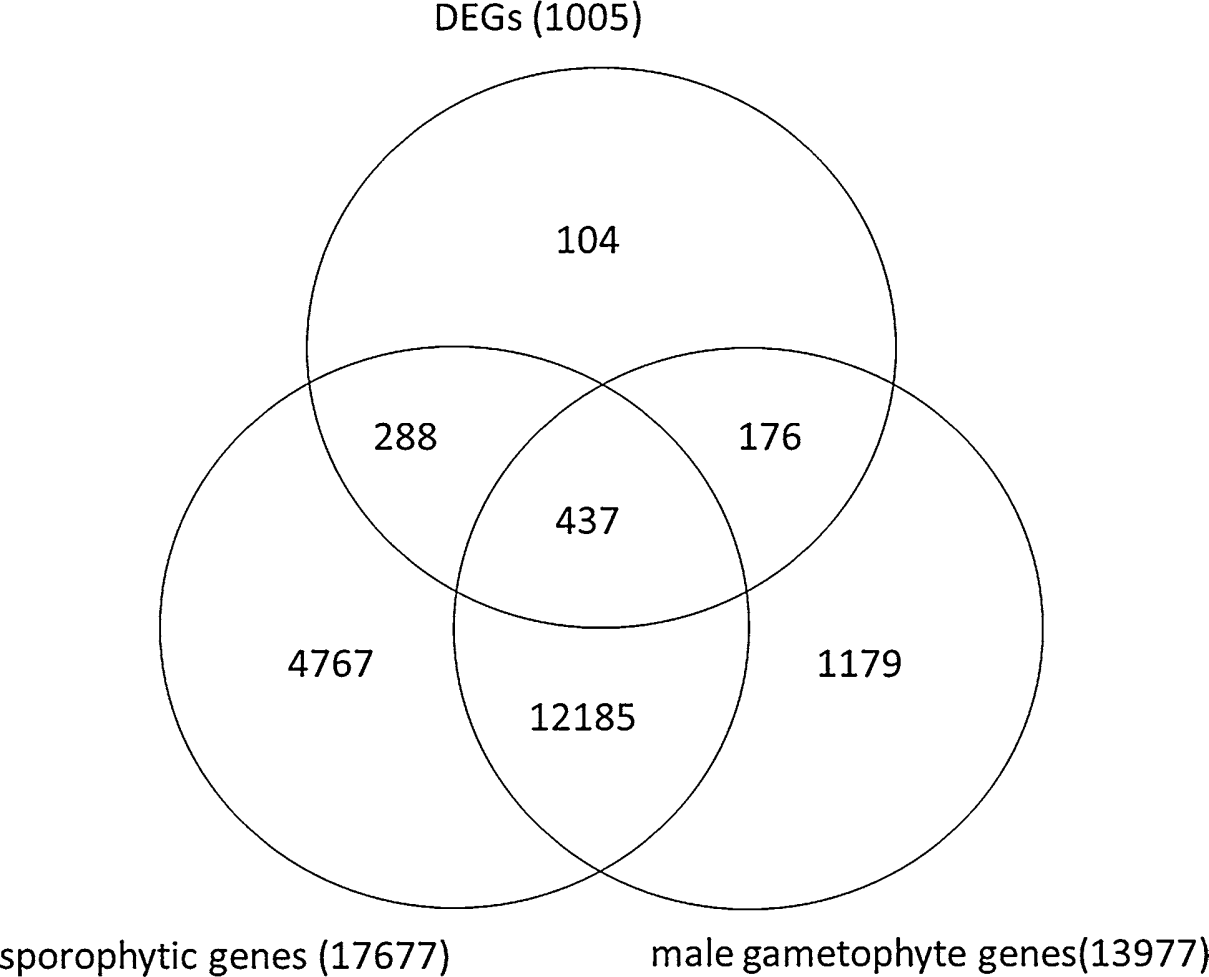
Comparison between differentially expressed genes (DEGs) and genes expressed in sporophytic and gametophytic tissues to identify non-pollen expressed genes (NPGs) of *B. oleracea*. A total of 17,677 sporophytic-expressed genes and 13,977 male gametophyte-expressed genes were detected in Honys’ research (Honys and Twell 2004). Of the DEGs, 725 genes were also sporophytic-expressed genes and 613 were also male gametophyte-expressed genes

**Fig. 6:**
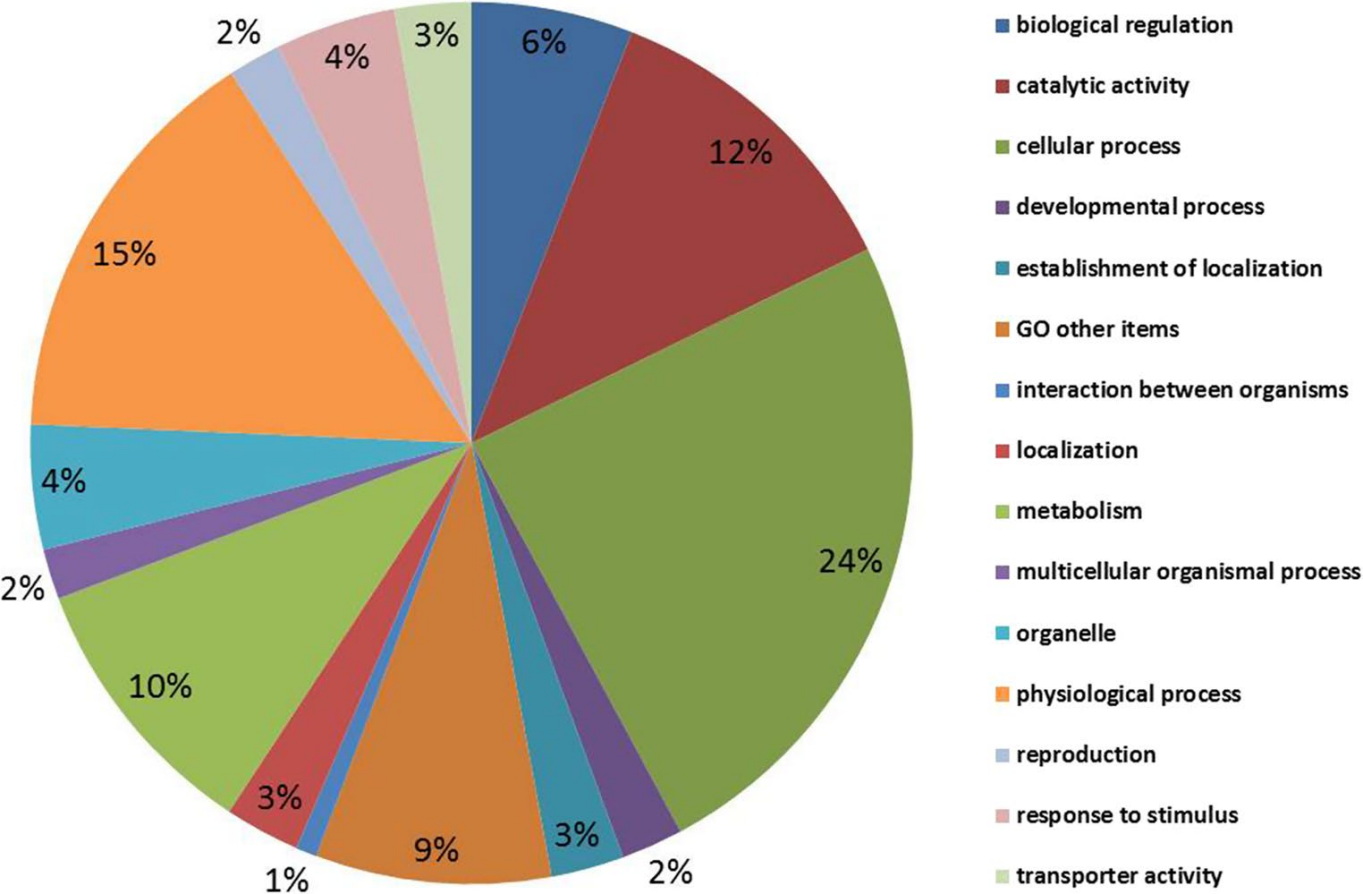
Gene ontology categorization of non-pollen expressed genes (NPGs) of *B. oleracea*. The NPGs were placed in 15 functional categories based on the MAS database

**Fig. 7:**
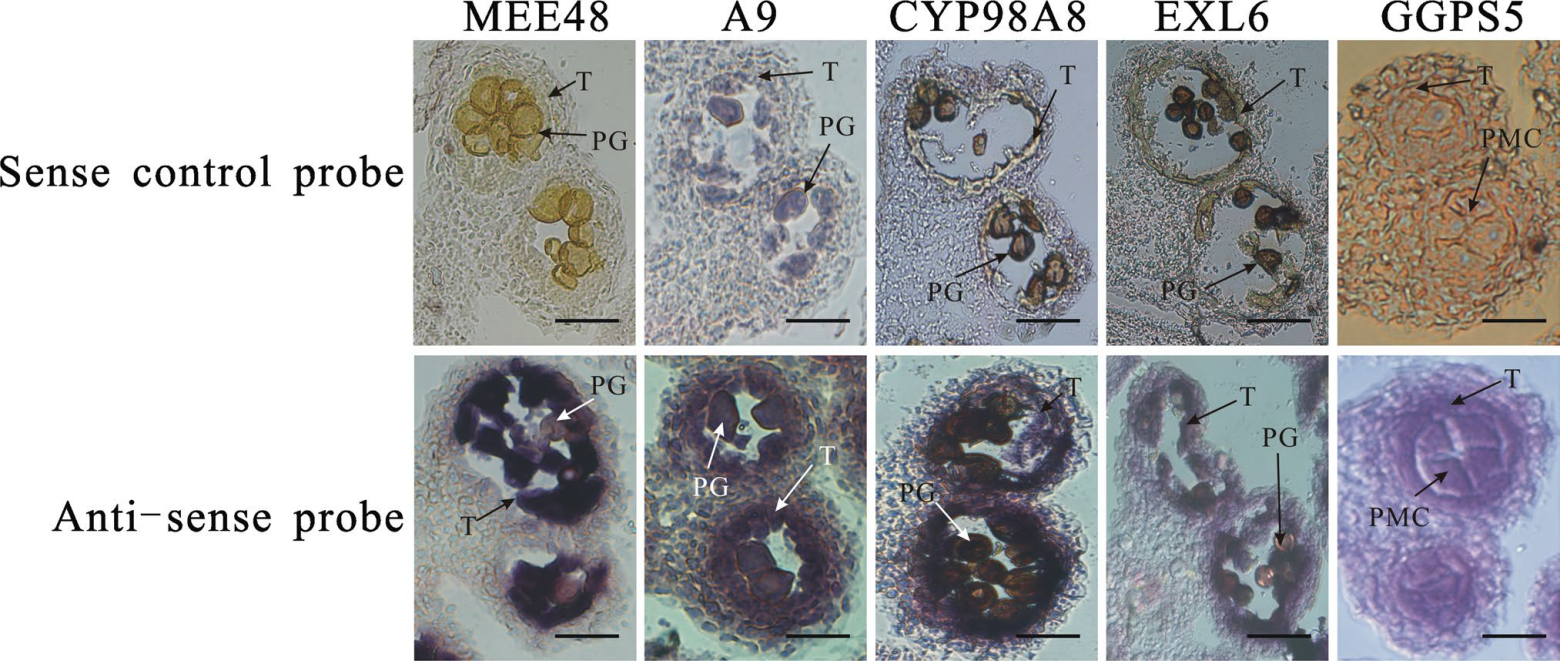
Five *Brassica oleracea* NPG mRNAs were localized by in situ hybridization to sections taken from developing anther locules of wild type (Col-0) *Arabidopsis* flowers. *Top line* using each of the five non-pollen expressed genes (NPGs) control sense probes. *Bottom line* using each of the five NPGs anti-sense probe. *Dark precipitate* indicates hybridization of the probe. *T* tapetum, *PG* pollen grain, *PMC* pollen mother cell. *Bar* = 70 µm

The correct spatiotemporal expression of genes in the anther is required for normal tapetum development. We clarified the sequential appearance and characteristics of the cytological defects of the four *B. oleracea* MS lines by comparing them with the wild-type (Fig. 1I). Light microscopy of the main anther developmental stages revealed that the abortive phenotypes appeared successively in the NiCMS line (Fig. 1I-7), the RGMS line (Fig. 1I-14), the OguCMS line (Fig. 1I-21), and finally in the DGMS line (Fig. 1I-28). We performed a TEM analysis to characterize the defective tapetum development in the four MS mutant lines (Fig. 1II). In the wild-type line, the sporogenous cells, which give rise to pollen, are visible within locules of sectioned anthers. Concentric rings of other cell types associated with pollen development and release are differentiated around the sporogenous cells during the sporogenesis cell stage (Fig. 1II-1). Sporogenous cells develop into microspore mother cells and four single distinguishable layers of anther wall and microsporangium could be observed during the microspore mother cell stage (Fig. 1II-2). Tetrads and tapetum with normal structures, as well as a single microspore tetrad with a central large nucleus, thick cytoplasm and abundant mitochondria develop during the tetrad stage (Fig. 1II-3).Vacuolated epidermal and endothelial cells, degenerating tapetum, and a free uninucleated microspore, containing a central nucleus, clear nuclear membrane, thick cytoplasm, and abundant plastids, appear in the post-tetrad stage (Fig. 1II-4). We compared the cytological features of the four male sterility types with those of wild-type. For each, we observed unique defective features. In the NiCMS line, the tapetal cells differentiated inconspicuously, with an indistinguishable middle layer at the sporogenesis cell stage (Fig. 1II-5). In the RGMS line, the tetrad aborted once it was formatted and the tapetal separated from anther wall at the microspore mother stage (Fig. 1II-6). In the OguCMS line, the tapetums were abnormally activated and thickened continuously when meiosis finished during the early tetrad stage (Fig. 1II-7). In the DGMS line, the morphology of the tapetum was not affected (Fig. 1II-8), as reported by (Lou et al. 2007). The development of microspores in the four MS lines was affected at different stages because of the abnormal tapetum development.

Genes with depressed expression levels became the focus of the research because the cytological observations indicated that MS lines were blocked by separate MS proteins. We hypothesized that a sequential developmental interruption model would clarify the gene expression sequence and be in accordance with the cytological results (Fig. 2). Based on the appearance point of the four MS phenotypes (NiCMS earlier than RGMS, RGMS earlier than OguCMS, OguCMS earlier than DGMS), the genes down-regulated only in the NiCMS lines were considered to express earlier than the genes down-regulated in both NiCMS and RGMS lines, and the genes down-regulated in both NiCMS and RGMS lines were considered to express earlier than the genes down-regulated in NiCMS, RGMS, and OguCMS lines. The latest expressing genes would be those that were down-regulated in all four MS lines. Genes involving in anther development mainly express in time series. The accumulation of products produced by early-expressing genes, such as transcription factors and secreted proteins., play important roles in expression of late-expressing genes (Wilson and Zhang 2009).

### Signal extraction of the microarrays

To reduce plant to plant and inflorescence branch-to-branch variations, we pooled the RNA obtained from three different plants. Pooling RNA before labeling has the advantage of reducing the variation due to biological replication and sample handling. Two replicate hybridizations were performed using pooled RNA from three different sterile or fertile plants and the labels were swapped for the second slide, with biological replicates. After the quantification of the signal intensities, the data were normalized to compensate for the nonlinearity of intensity distributions and differences in probe labeling (Fig. 3). Using a signal/noise value >5 to select for positive signals, we detected 12,838 positive signals both in NiCMS control samples and NiCMS samples; 13,037 positive signals both in RGMS control samples and RGMS samples; 13,083 positive signals both in OguCMS control samples and OguCMS samples; and 11,581 positive signals both in DGMS control samples and DGMS samples. The reproducibility was determined by calculating the coefficient (R^2^) of the Log_2_ normalized signal values of all detected signals. The R^2^-value between replicas were as follows: 0.9847 for the NiCMS control (Fig. 3a) and 0.9808 for the NiCMS samples (Fig. 3b); 0.9754 for the RGMS control samples (Fig. 3c) and 0.9663 for the RGMS samples (Fig. 3d); 0.9708 for the OguCMS control samples (Fig. 3e) and 0.9834 for the OguCMS samples (Fig. 3f); and 0.9772 for the DGMS control samples (Fig. 3g) and 0.9738 for the DGMS samples (Fig. 3h). The consistency of the two slides, together with the large number of detectable genes, indicates the feasibility of using the Agilent Arabidopsis 2 Oligo array to analyze the *B. oleracea* transcriptome.

### Microarray analysis

An Agilent Arabidopsis 2 Oligo array was used to compare gene expression profiles of the four *B. oleracea* MS lines to their MF control lines. The array contained 21,500 probes for genes or transcripts of *Arabidopsis*. Hybridizations of two replicates were performed using pooled RNA from each of three different sterile or fertile plants, and labels were swapped for the second slide with biological replicates. A signal/noise value of >5 was used to select positive signals; this identified 12,837, 13,036, 13,082, and 11,580 genes in NiCMS, RGMS, OguCMS, and DGMS lines, respectively. After combining these four datasets 13,984 genes (65.0 %) were detected as positive signals in at least one of the MS lines. This percentage of identified genes was similar to previous research that identified 14,660 (64.5 %) genes in six organs and structures, including the inflorescences, at different *Arabidopsis* floral stages (Zhang et al. 2005). Genes with differential mRNA abundance levels (ratios >3, or <0.33) in the two replicate slides were selected for further analysis. To confirm the microarray profiling data, nine genes were randomly selected for semi-quantitative RT-PCR analyses, and their expression patterns were found to be consistent with the microarray results (Fig. 4, Supplementary Table S1).

There were 544, 338, 526, and 209 down-regulated genes, and 5, 45, 151, and 15 up-regulated genes detected in the NiCMS, RGMS, OguCMS, and DGMS lines, respectively (Supplementary Table S2 and S3). After redundant genes were merged, 838 DEGs were down-regulated (group 1) and 188 up-regulated (group 2) in at least one of the four MS lines. The overlap of group 1 and group 2 contained 21 DEGs, including the two tapetum-related genes *TDF1* and *MYB 103*. These genes play vital roles in tapetum synthesis and degeneration, and the sporopollenin monomer biosynthesis process (Higginson et al. 2003). These genes were down-regulated in the NiCMS line but up-regulated in RGMS and OguCMS lines. Following the removal of these 21 DEGs, 1,005 non-redundant DEGs were identified in this research.

### Identification of tapetum-specific genes

The tapetum has been studied in many kinds of plants using MS defects, and, due to the difficulty of tapetum isolation, only four genes have been reported to be tapetum specific by MS mutant gene cloning (Suwabe et al. 2008). Therefore, we developed an approach to identify tapetum-specific genes on a large-scale, and at the developmental stages in which they are expressed. Fortunately, the separation of male gametophytes is easy, and numerous male gametophyte-specific genes have been identified from multiple MS mutants and global transcriptome analyses.

To identify genes specifically involved in tapetum development, we removed genes known to be expressed in sporophytic tissues based on the findings of Honys and Twell 2004. Their research identified 17,677 sporophytic genes, of which 725 genes overlapped with the 1,005 DEGs identified in our research (Supplementary Table S4). This left 280 DEGs thought to be expressed specifically in the anthers. Furthermore, 176 male-gametophyte expressed DEGs identified by (Honys and Twell 2004) were removed from the 280 DEGs specifically expressed in the anthers, leaving 104 NPGs (Fig. 5). As the four MS lines had tapetums aborted at successive developmental stages, we were able to identify 104 NPGs as anther wall-specific genes, the vast majority of which were considered tapetum-specific genes because their anther walls developed normally with the exception of the distinct abortion of the tapetums (Table 2). We cannot completely rule out that some genes expressed from other tissues are included in the 104 NPGs, although this probability is very low.

Male sterility genes block the developmental pathways of anthers at a certain stage, and lead to abnormal anther development. Because each of the four MS lines showed distinct tapetum abortive phenotypes, and their abnormal characteristics appeared successively during anther development, we could deduce the expression sequence of the NPGs by their expression patterns. The expression sequence of the 55 NPGs could be determined according to the sequential appearance of the tapetum abortive phenotypes (Table 2). First, 28 NPGs that were only down-regulated in the NiCMS line were expressed, followed by seven NPGs down-regulated in both the NiCMS and RGMS lines, then 16 NPGs down-regulated in the NiCMS, RGMS, and OguCMS lines, and finally four NPGs down-regulated in the four MS lines were expressed. It is thought these 55 NPGs constitute the main stream of tapetum development, while the remaining 49 NPGs belonged to bypass ways which also play roles in tapetum development. These results showed that the tapetum development is strongly correlated with gene expression patterns and anther developmental timing. In the eight previously reported tapetum-specific genes, five (*ACOS5*, *A6*, *LTP12*, *LAP5*, and *A9*) were detected in our filtered microarray results, although the other three tapetum-specific genes (*TSM1*, *TAP35*, and *TAP44*) were detected in our microarray analysis at variable expression levels in the four MS lines. Because of the strict filter these three tapetum-specific genes were not included in our set of 104 NPGs. There were many noticeable features in the up-regulated genes, with 13 NPGs up-regulated in the OguCMS line, five NPGs up-regulated in both the RGMS and OguCMS lines, and one NPG up-regulated in both the RGMS and DGMS lines (Table 2). This is consistent with the cytological observations of different abnormal tapetum development in the four MS lines.

The tapetum developmental network is regulated by many genetic pathways (Wilson and Zhang 2009). MS mutants occurring at different developmental stages lead to abnormal downstream reactions, including altered tapetum structure and gene expression patterns. These changes are evoked by the presence of MS-associated proteins (Fujii et al. 2010). Only a few previous studies have analyzed gene expression patterns in *B. oleracea* MS lines (Kang et al. 2008). These studies compared anther gene expression profiles in MS lines with their corresponding fertile lines, allowing for the preferentially expressed anther genes to be identified. Despite their clear contribution to anther developmental pathways, downstream expressed anther wall-specific genes have been overlooked because of the removal of male gametophyte-specific expressed genes.

### Function of genes arrested by the four types of *B. oleracea* MS lines

The distribution of the NPGs was determined in the gene ontology data set (MAS 3.0, http://bioinfo.capitalbio.com/mas3/) and found to cover virtually all functional categories (Fig. 6). The classification of functional categories revealed that some were enriched in DEGs that had reduced expression levels, including structural molecules, transporters, and physiological processes. These categories are associated with metabolic activities that are dynamic in the tapetum, suggesting a positive role of the tapetum in the regulation of metabolic functions. As we are interested in the genetic mechanism of tapetum abortion in the four MS lines, genes specifically expressed in the tapetum were further analyzed to identify tapetum abortive phenotypes.

Special attention was paid to abortive mechanisms leading to tapetum dysfunction by gene regulation networks within the 1,005 DEGs in any of the MS lines. According to previous studies, 22 DEGs were proven to be related to tapetum development (Supplementary Table S6). Among these 22 genes, 11 of the DEGs were reported to play important roles in tapetum development (*ULT2*, *TDF1*, *PGA4*, *PAB3*, *TKPR2*, *PAB5*, *SHT*, *ACT12*, *LAP6, HMA4*, and *ATA20*). More importantly, the other 11 DEGs were identified as NPGs in this study (*LTP12*, *CYP703A2*, *CYP704B1*, *LAP5*, *ACOS5*, *ABCG26*, *MYB103*, *MYB99*, *WBC27*, *ATBHLH089*, and *ATBHLH091*) (Table 2). To determine the expression patterns of NPGs, we compared them with other mutant transcriptomes that have been analyzed by bioinformatics filtering. The results showed that 27 (26 %) NPGs were also detected in an anther-specific expressed gene set (Xu et al. 2014), 32 (31 %) NPGs were detected in a stamen-specific expressed gene set that excluded pollen-specific expressed genes (Ma et al. 2012). To validate microarray results in anthers, we performed in situ hybridization using five randomly chosen NPG-derived probes and developing wild-type *A. thaliana* flowers (Fig. 7). We used *MEE48*, *A9*, *CPY98A8*, and *EXL6* probes that hybridized with anthers when they had developed to the uninucleated microspore stage, respectively. *GGPS5* probes were hybridized with anthers when they developed to the pollen mother cell stage. No hybridization signal was observed in locules with microspores and tapetum using any of the NPG sense control probes. However, NPG anti-sense probes resulted in varying degrees of hybridization to the tapetum. These results showed that our data set, which was generated by bioinformatics filtering, was reliable. Therefore, the NPGs can be seen as potential tapetum-specific expressed genes.

## Conclusions

In this study, four different types of *B. oleracea* MS lines, covering the whole of anther development, were sampled for microscopy and microarray analysis. A systematic study of the tapetum developmental and molecular phenotypes of the four *B. oleracea* MS lines was performed using the Agilent *Arabidopsis* 2 Oligo array, which contains 25,000 probes to known or predicted genes. This is the first large-scale trial to explore the spatial and temporal expression patterns of tapetum-specific gene in *B. oleracea* MS lines. The results revealed that most tapetum-specific genes were expressed in a stage-specific manner.

The most significant phenotype of the four *B. oleracea* MS lines is that the anther walls are normally developed, with the exception of the distinct tapetum abortive phenotypes. These abnormal characteristics appeared successively during anther development (Fig. 1). The abortive phenotypes first appears in NiCMS, then in RGMS, followed by OguCMS, and finally in DGMS. A sequential developmental interruption model was proposed to clarify the expression order of the DEGs in plants. MS-associated proteins might directly or indirectly regulate some of the genes involved in tapetum synthesis and degeneration, and then block the expression of a large number of genes involved in normal microspore development. Eliminating sporophytic-and male gametophyte-specific expressed genes identified in the NPGs as anther wall-specific genes, resulted in the majority of remaining genes being tapetum specific. We defined the expression sequence of 55 NPGs based on the sequential developmental interruption model and determined that they are involved in structural molecules, transporters, and physiological processes related to cell wall modification and catalytic activities. The categories suggested the metabolic role of the tapetum in the regulation of anther development. These results outline a methodology to retrieve information on hard to isolate tissues through the comparison of global expression with gene expression in easily obtained mutants. The relationship of anther-expressed genes can be clarified by comparing the sequential cytological appearance of defects in multiple independent MS lines with the same tissues in the wild-type.

## Electronic supplementary material

Below is the link to the electronic supplementary material.

Supplementary material 1 (XLSX 10 kb)

Supplementary material 2 (XLSX 107 kb)

Supplementary material 3 (XLSX 23 kb)

Supplementary material 4 (XLSX 111 kb)

Supplementary material 5 (XLSX 10 kb)

Supplementary material 6 (XLSX 13 kb)
